# A Comparison of Capillary and Venous Blood Haematocrits of Pregnant Women in Nigeria: The Impact on Diagnosis and Prevalence of Anaemia in Pregnancy

**DOI:** 10.1155/2014/467056

**Published:** 2014-01-28

**Authors:** Cyril Chukwudi Dim, Emmanuel Onyebuchi Ugwu, Ugochukwu Bond Anyaehie, Kingsley Chukwu Obioha

**Affiliations:** ^1^Department of Obstetrics and Gynaecology, University of Nigeria Teaching Hospital, PMB 01129, Enugu 400001, Nigeria; ^2^Department of Physiology, College of Medicine, University of Nigeria, Enugu Campus, PMB 01129, Enugu 400001, Nigeria

## Abstract

*Background*. Volume of red cells in capillary blood varies from that of venous blood. The magnitude of this variation as well as its impact on the diagnosis of anaemia in pregnancy needs to be studied. This study demonstrates the disparity between capillary and venous PCV in pregnancy. *Objectives*. To determine whether capillary blood PCV (cPCV) differed from venous blood PCV (vPCV) of normal pregnant women in Enugu, Nigeria, and its effect on diagnosis and prevalence of anaemia. *Methods*. PCV was estimated using pairs of venous and capillary blood samples from 200 consecutive pregnant women at the Antenatal Clinic of University of Nigeria Teaching Hospital, Enugu, Nigeria. *Results*. Participants' cPCV (median = 34.0%, IQR = 31.0–35.8) was significantly lower than their vPCV (median = 34.0%, IQR = 32.0–37.0) (*Z* = −6.85, *P* < 0.001). However, women's cPCV had strong positive correlation with their vPCV (*r* = 0.883, *P* < 0.001). The prevalence of anaemia among participants using capillary and venous blood was 33.5% (67/200) and 28.0% (56/200), respectively (O.R = 1.3 (CI 95%: 0.85, 1.98),  *P* = 0.233). *Conclusions*. Capillary blood PCV was lower than vPCV among pregnant women in Enugu, Nigeria. Nevertheless, the prevalence of anaemia derived from cPCV did not differ significantly from that of vPCV.

## 1. Introduction

Pregnancy is a physiological condition which is characterized by lots of systemic changes necessary to support the growth of the fetus—these changes include increases in blood volume, red cell mass, and plasma volume [1]. Though most pregnancies are normal, complications often arise causing varying degrees of maternal and perinatal morbidity and mortality. An important complication of pregnancy globally and particularly in developing countries is anaemia [2–5] which is defined as having below normal values for the total volume of red blood cells, the number of normal red blood cells, or the amount of hemoglobin in these cells [6]. Likewise, the total volume of blood made of red blood cells, otherwise referred to as the haematocrit (Hct) or packed cell volume, (PCV) is often used to screen for anaemia in pregnancy as part of secondary preventive health strategy in prenatal care, and a value of less than 33% is considered as anaemia [3].

Furthermore, blood samples for assessment of PCV are collected from the capillaries or veins, and the results got are often accepted without regard to the source of the specimen. For adults in our environment, the capillary blood samples especially from finger pricks are often used for PCV estimation probably because it is easier and faster. Nevertheless, because of the peculiarities of blood and capillary microcirculation, the volume of red cells in capillary blood varies from that of the venous blood [7]. This disparity between capillary and venous blood PCV has been reported in a few studies [8–10]. But, there is no accessible report of this disparity in a population of pregnant women. The concern is heightened by the fact that PCV results from both capillary and venous blood are used interchangeably in the study environment, and diagnosis of anaemia often made without recourse to the source of blood used for the estimation. This study therefore aimed to determine the disparity between capillary (cPCV) and venous blood PCV (vPCV) in normal pregnancy. It is hoped that the study findings will improve the diagnosis and follow-up of anaemia in pregnancy by encouraging uniformity of blood source for PCV estimations.

## 2. Materials and Methods

### 2.1. Study Center

The study was carried out at the Antenatal Clinic (ANC) of the University of Nigeria Teaching Hospital (UNTH), Enugu, Nigeria. The hospital is a tertiary health care institution owned by the federal government of Nigeria. It offers both primary and specialized health care services to residents of Enugu state and environs. The ANC is an obstetrician-led prenatal care which holds every weekday. Routine haematinics are offered to all pregnant women during antenatal care.

### 2.2. Study Area

Enugu state is one of the five states in southeast geopolitical zone of Nigeria. It lies within the West African rain forest region (latitudes 5°  55′′ and 7°  10′′ north and longitudes 6°  50′′ and 7°  55′′ east), through a land area of approximately 8000 km^2^ [11]. The inhabitants are predominantly Igbos with a population of over 3 millions [12]. The prevalence of poverty in the state is about 60% which implies all the associated problems of low incomes, and poor education and health [11]. Further details of the study area have been described in a recent study [13].

### 2.3. Study Design

The study was a cross-sectional analytical study of 200 consecutive consenting normal pregnant women attending the ANC of the UNTH, Enugu, Nigeria, from 15 May 2012 to 25 June 2012. All normal singleton pregnant women receiving care at the clinic within the study period were eligible for the study. Exclusion criteria were being unsure of date having medical illness in pregnancy such as diabetes mellitus and HIV infection and obstetrics complications including preeclampsia and antepartum haemorrhage.

The study started after approval by the Ethical Committee of the UNTH, Enugu. Group counselling about the study was administered to pregnant women each weekday, before the start of the antenatal clinic. Those who expressed interest were screened for eligibility after which individual counselling was offered and written informed consent was obtained. Each selected woman served as her own control. Participants' recruitment continued till the targeted sample size was achieved. Based on a previous study among pregnant women in Enugu, Nigeria, which found a standard deviation of the mean venous blood PCV of 1.41% among HIV-seronegative controls [14], the sample size of 200 used for this study was adequate to identify a minimum meaningful mean PCV difference of 0.49% at 95% confidence level and 90% power.

### 2.4. Data Collection and Analysis

Participants' sociodemographic characteristics including age, educational status, marital status, and parity were sought by trained research assistants (medical interns) using semistructured questionnaires.

The thumb (palmar surface of distal phalanx) and the cubital fossa of the preferred arm were cleaned using cotton wool soaked with 70% alcohol [6]. Then, a quick stabpuncture was made with a sterile lancet on the thumb of the chosen arm and the freely flowing blood was collected in two plain standard-sized heparinised capillary tubes without squeezing the thumb, until each was 75% full [6]. Afterwards, 0.5 mL of blood was collected from the antecubital vein of the preferred arm with a sterile 2 mL syringe. The blood was transferred immediately to 2 other plain heparinised capillary tubes. Each capillary tube containing blood sample was sealed at the inferior end with an appropriately coloured clay sealant (red for venous blood and blue for capillary blood) and centrifuged for 5 minutes using microhaematocrit centrifuge. The PCV for each capillary tube was read immediately after centrifuging, with a microhaematocrit reader. The mean value for each category (capillary or venous) was recorded on a data sheet designed for the study. The result of the venous PCV for each study participant was issued to her on the same day and those whose results indicated anaemia were promptly referred to their attending obstetricians.

Data analyses were both descriptive and inferential at 95% confidence level using SPSS version 15 for windows. Kolmogorov-Smirnov test was used to assess normality of continuous data. Associations were compared using Wilcoxon Signed-Ranks Test and Spearman's Correlation Coefficient for continuous data and cross tabulation for categorical data. Results were presented using simple percentages and tables as appropriate. Associations between variables were shown using *P* values, odd ratios, and confidence intervals. A *P* value of less than 0.05 was considered as statistically significant.

Primary outcome measure was the difference between capillary and venous blood PCV of healthy pregnant women in Enugu, Nigeria, while the secondary measure was the prevalence of anaemia in pregnancy using cPCV and vPCV. Anaemia in pregnancy was defined as in the introduction [3].

## 3. Results

Two hundred normal pregnant women participated in the study. Their mean age was 30.1 ± 5.05 years (range = 19–45). A majority (99.0%) of participants were married. Ten (5.0%) women had primary education and 42 (21.0%) had secondary education, while 148 (74%) women had tertiary education. The mean parity was 1.3 ± 1.51 (range = 0–6). Eighty-eight women (44.0%) were nullipara, 44 (22.0%) primipara, 60 (30.0%) multipara, and 8 (4.0%) grandmultipara. One hundred and eighteen (59.0%) women had gestational ages of 28 weeks and above. Further details of participants' characteristics are shown in Table 1.

The capillary blood PCV (cPCV) ranged from 20.0 to 44.0% (mean = 33.5 ± 3.57) while the range for venous blood PCV (vPCV) was 21.0–44.0% (mean = 34.3 ± 3.74). Both cPCV and vPCV values were nonnormally distributed (*P* < 0.05). However, Wilcoxon Signed-Ranks Test showed that participants' capillary blood PCV (median = 34.0%, IQR = 31.0–35.8) was significantly lower than the vPCV (median = 34.0%, IQR = 32.0–37.0) (*Z* = −6.85, *P* < 0.001, *r* = 0.48).

**Figure 1:**
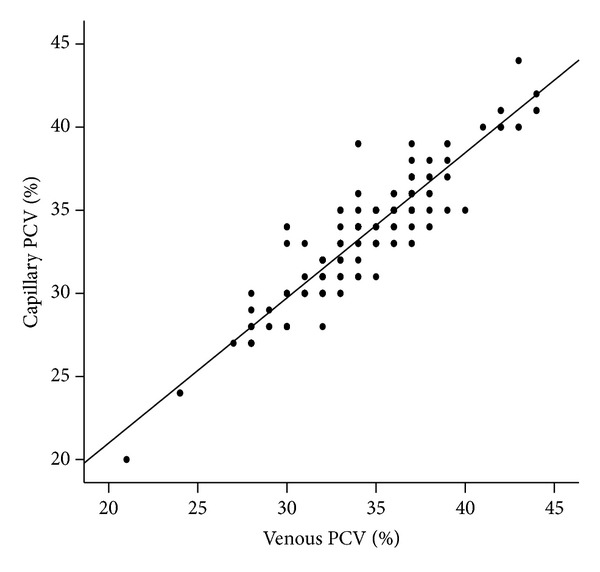
Correlation between capillary PCV and venous PCV.

Out of the 200 PCV pairs, cPCV was higher than vPCV in 18 (9.0%) women, equal in 68 (34.0%) women, but less than vPCV in 114 (57.0%) women. The difference between each PCV pair (cPCV minus vPCV) ranged from −5 to +5% (mean = −0.83 ± 1.54). Furthermore, as shown in Figure 1, the cPCV values of participants had strong positive correlation with their venous values (*r* = 0.883, *P* < 0.001).

The prevalence of anaemia among participants using capillary and venous blood was 33.5% (67/200) and 28.0% (56/200), respectively. The observed difference was not statistically significant (O.R = 1.3 (CI 95%: 0.85, 1.98),  *P* = 0.233)—details are shown in Table 2.

Among participants whose GA were 28 weeks and above, the prevalence of anaemia using capillary blood was 28.8% (34/118) while that of venous blood was 24.6% (29/118) (O.R = 1.2 (CI 95%: 0.70, 2.22),  *P* = 0.462). Similar association was observed among women whose GA were less than 28 weeks (Table 2). With respect to the difference in the prevalence of anaemia between capillary and venous blood (Table 3), the widest disparity (7.3%) was observed among women whose GA were less than 28 weeks.

## 4. Discussion

This study showed that over 40% of pregnant women in the study area belong to the 26–35-year age group which is not surprising since the average age at first pregnancy at the study center was 26.2 years [15]. Also, the distribution of marital status of participants was consistent with previous report [16]. A majority of study participants had tertiary education which may suggest that women with higher education seek antenatal care at the study center; this finding has also been observed in earlier studies [16, 17]. This assumption may be correct because higher education is expected to drive quality health seeking behaviour.

This study found that the cPCV was significantly lower than vPCV which is consistent with the report that red cell volume in capillary blood is lower than venous blood [6]. It is however not consistent with studies among nonpregnant adults in Norway and China which reported higher cPCV when compared to vPCV [8, 10]. The small sample sizes used in both studies were likely to have affected their precision. Unfortunately, an extensive literature search did not identify any related study among pregnant women with which this study's results can be compared.

The reasons for the reduced capillary red cell volume in this study were likely due to the inherent mechanisms within the human microvasculature which impact the dynamics of blood flow—one of the implicated mechanisms is the “screening effect” which implies the prevention of RBC entry into the small capillary due to insufficient deformation [18]. Other mechanisms include the uneven distribution of RBCs to daughter branches at microvascular bifurcations (plasma skimming), the axial accumulation of erythrocytes in the small blood vessels, and the difference in travelling speed between cells and plasma in the microcirculation (Fahraeus effect) [7, 19, 20]. These mechanisms are affected by vascular smooth muscle activity [21]. Therefore, since normal pregnancy is associated with a reduction in vascular resistance, it is likely that the magnitude of the cPCV and vPCV disparity observed in this study will differ from that of nonpregnant population. There is a need for further studies in this direction. In this study, the maximum variability between capillary and venous PCV pairs was 5%. This magnitude of variability can impact patients' management especially as regards the decision to transfuse blood or blood products. It is therefore suggested that clinical units should define the blood source that should be used for their patients' management so as to ensure consistent and reproducible results. Where such guideline does not exist, serial PCV monitoring in patients such as during antenatal care should employ a consistent blood source for the same reason. Likewise, it has also been suggested that, in cases where moderate to severe anaemia is suspected clinically, vPCV should be used as a matter of policy because it gives more accurate result [6].

Furthermore, the study showed a disparity between the prevalence of anaemia recorded from cPCV and vPCV, though the observed difference was not statistically significant. In view of this possible magnitude of variability, comparison of results of studies on anaemia in pregnancy should pay attention to the sources of blood samples. It is interesting that the prevalence of anaemia (all sources) increased among women at GA of less than 28 weeks but reduced among those in 3rd trimester (≥28 weeks). This observation might be due to the effect of physiological anaemia of pregnancy which is known to be pronounced in 2nd trimester [1].

Also, with respect to the prevalence of anemia in pregnancy, the difference between the values got from cPCV and vPCV was higher for women of GA less than 28 weeks when compared with women in 3rd trimester. The reason for this obvious disparity was not clear, but it might still be related to, the varying peripheral vascular resistance which falls in early pregnancy but increases slowly in 3rd trimester until term [22].

The study was limited by its base in one tertiary health facility which might affect its generalization to all pregnant women in Enugu, Nigeria. On the other hand, the strength of the study lies in the fact that it is the first reported effort in Nigeria to demonstrate the disparity between capillary and venous PCV, irrespective of the study population. It also highlights the need for care givers to be conscious of the source of blood used in the determination of PCV for pregnant women.

## 5. Conclusion

This study has demonstrated that the packed cell volume from capillary blood was significantly lower than that of venous blood of antenatal women in Enugu, Nigeria. Likewise, the prevalence of anaemia in pregnancy derived from PCV of capillary blood was higher than that of venous blood by about 20%, though the difference was not significant. In view of these observations, maternity units are encouraged to use venous blood for PCV estimation or at least be consistent with any chosen source of blood for PCV estimation.

## Figures and Tables

**Table 1:** Distribution of participants' characteristics.

Maternal characteristic (*n* = 200)	Subcategory	Frequency (%)
Age groups (years)	16–20	5 (2.5)
	21–25	33 (16.5)
	26–30	65 (32.5)
	31–35	76 (38.0)
	36–40	12 (6.0)
	41–45	9 (4.5)

Tribe	Igbo	191 (95.5)
	Yoruba	2 (1.0)
	Hausa	0 (0.0)
	Others	7 (3.5)

Gestational age groups (weeks)	<28	82 (41.0)
	≥28	118 (59.0)

**Table 2:** Association between prevalence of anaemia and source of blood.

GA category (weeks)	Source of blood	Anaemia in pregnancy		*P* value	O.R (CI: 95%)
		Yes (%)	No (%)		
All GA	Capillary	67 (33.5)	133 (66.5)	0.233	1.3 (0.85, 1.98)
	Venous	56 (28.0)	144 (72.0)		—
<28	Capillary	33 (40.2)	49 (59.8)	0.331	1.4 (0.73, 2.60)
	Venous	27 (32.9)	55 (67.1)		—
≥28	Capillary	34 (28.8)	84 (71.2)	0.462	1.2 (0.70, 2.22)
	Venous	29 (24.6)	89 (75.4)		—

**Table 3:** Disparity between anaemia from capillary and venous blood PCV.

Gestational age groups (weeks)	Prevalence of anaemia (%)		Difference b/w anaemia prevalence from cPCV and vPCV (%)	Percentage increase in prevalence* (%)
	cPCV	vPCV		
All GA	33.5	28.0	5.5	19.6
<28	40.2	32.9	7.3	22.2
≥28	28.8	24.6	4.2	17.1

* = (cPCV − vPCV) × 100/vPCV.
